# Revealing How Topography of Surface Microstructures Alters Capillary Spreading

**DOI:** 10.1038/s41598-019-44243-x

**Published:** 2019-05-24

**Authors:** Yaerim Lee, Naoto Matsushima, Susumu Yada, Satoshi Nita, Takashi Kodama, Gustav Amberg, Junichiro Shiomi

**Affiliations:** 1 Department of Mechanical Engineering, The University of Tokyo, Bunkyo-ku, Tokyo, Japan; 2 Department of Mechanics, Linné Flow Centre, The Royal Institute of Technology, Stockholm, Sweden; 3 Södertörn University, Stockholm, Sweden

**Keywords:** Mechanical engineering, Fluid dynamics

## Abstract

Wetting phenomena, i.e. the spreading of a liquid over a dry solid surface, are important for understanding how plants and insects imbibe water and moisture and for miniaturization in chemistry and biotechnology, among other examples. They pose fundamental challenges and possibilities, especially in dynamic situations. The surface chemistry and micro-scale roughness may determine the macroscopic spreading flow. The question here is how dynamic wetting depends on the topography of the substrate, i.e. the actual geometry of the roughness elements. To this end, we have formulated a toy model that accounts for the roughness shape, which is tested against a series of spreading experiments made on asymmetric sawtooth surface structures. The spreading speed in different directions relative to the surface pattern is found to be well described by the toy model. The toy model also shows the mechanism by which the shape of the roughness together with the line friction determines the observed slowing down of the spreading.

## Introduction

How do the detailed properties of a dry surface influence the speed at which a liquid spreads over it? From splashing around solid objects in water, to the way plants and insects imbibe water and moisture, surface tension and wetting of solid surfaces are essential^1–3^. In technology, miniaturization in chemistry and biotechnology has generated strong interest in wetting phenomena, as a means to control and manipulate small samples and droplets^4,5^. Inspired by these, droplets impacting or moving over dry surfaces have recently received considerable attention^6–10^.

Such motions may be controlled by the microscopic surface structure, both in terms of geometric features and variations in surface energy. An additional complexity is introduced when the microscopic surface structure is highly anisotropic. In nature this is found for instance on the feet of water striders, which are covered with hydrophobic hairs, the orientation of which is important for the locomotion of the insect^11,12^. In technology there are many situations where it is desirable to control the motion of droplets, and anisotropic surfaces can be prepared to achieve this, see for instance^13,14^.

The understanding of the physics of a moving contact line, the intersection of a fluid interface and a solid, is still evolving (See the recent reviews^15–17^). It is a classical problem in fluid dynamics that the equations for viscous flow produce a non-integrable singularity of the viscous stress at the wall^18^. The root of this problem is that micro- and nano-scale processes at the contact line may have macroscopic effects and determine the spreading speed. For the late spreading of a droplet towards equilibrium, the classical Tanner’s law^19^ introduces an adhoc cutoff length and assumes that viscous dissipation in the bulk liquid near the contact line provides the dominant resistance.

In the generic situation of a droplet that is placed on a dry surface, spreading due to surface tension and the wetting properties of the surface, it has in many situations been found experimentally^20–23^ that the fast early spreading is dominated by inertia and can be surprisingly insensitive to the substrate properties. Very recently, experiments have also shown that the early spreading may have a first viscous regime, also for not very viscous liquids^24^. The final stages of wetting, when the droplet dynamics has become quasi-static and the contact angle is close to its static value, are generally described well by Cox-Voinov or Tanner’s law, indicating that the viscous flow in the wedge shaped region near the contact line determines the speed^25,26^. See for instance Kant *et al*.^27^ for a study of spreading over a complex substrate geometry in this parameter range. Here we will be concerned with the rapid initial motion which is further from equilibrium.

Approaching wetting from the molecular point of view, another line of research discusses the resistance to wetting in terms of a non-hydrodynamic energy dissipation at the contact line^28–33^, that can be empirically quantified in terms of the contact line friction parameter *μ*_*f*_ (in Pascal seconds, Pa s)^28^,

1 $$\begin{array}{c}w \sim \,{\mu }_{f}{U}^{2}\end{array}$$

where *U* is the contact line speed, and *w* the energy dissipation rate associated with molecular processes at the contact line, per unit length. In the Molecular Kinetic Theory (MKT)^28–30^, dynamic wetting is described as an activated process on the molecular scale, and the line friction *μ*_*f*_ is given a phenomenological interpretation on the molecular scale. On a macroscopic scale, the consequence of the presence of contact line friction is that there is a relation between the dynamic contact angle and the contact line speed. Viewed as a macroscopic phenomenological parameter, *μ*_*f*_ may be connected to the ‘Hocking parameter’^34,35^, which is the assumed constant of proportionality between the deviation of the dynamic contact angle from its equilibrium value and the contact line speed.

Values of *μ*_*f*_ can be obtained from straightforward spreading experiments^36^ in conjunction with numerical simulations. The recent studies present independent measurements of line friction values^33,37^. Vo and Tran^33^ clarified the dependency of the contact line friction on the viscosity both of the droplets and the surrounding oils. Xia and Steen^37^ report measurements using water – glycerol mixtures on silicon substrates treated to be partially wetting and to have low contact angle hysteresis. The mobility in their case is the constant of proportionality between the deviation of dynamic contact angle from the static value, and the contact line speed. This can readily be translated into the line friction *μ*_*f*_ used here. We have not performed tests on the exact same surfaces as theirs, but note that the numbers they report give values of *μ*_*f*_ in the range 0.17–0.53 Pa s, increasing with increasing glycerol content, similar to our values. Recently contact line friction was also measured for very viscous polymer melts^38^, which reveals a line friction proportional to the bulk viscosity, with a constant of proportionality greater than one so that the contact line friction is always greater than the bulk viscosity.

We will not be concerned here with the molecular origin of *μ*_*f*_, but rather consider it as a material parameter, characteristic of the combination of the particular liquid and the solid surface. We are considering however the role of line friction in the fluid dynamics of wetting, and in particular how the influence of the details of the substrate properties and geometry on dynamic wetting can be understood in terms of this.

In spreading over rough surfaces, it was found that the effective line friction coefficient *µ*_*f*_ that characterizes the macroscopic spreading can be written as

2 $$\begin{array}{c}\,{\mu }_{f}=\,{\mu }_{f,flat}S\end{array}$$

where *S* denotes the ratio of the total wet surface area of the structure to the projected footprint area, and *μ*_*f*,*flat*_ is the line friction obtained on a (smooth) perfectly flat surface of the same material^36^. The total line friction could thus be separated into one surface-chemistry factor *μ*_*f*,*flat*_ and one purely geometric characterization of the roughness, *S*. In experiments on droplets impacting structured surfaces^39,40^, it was found that the maximum radius of the droplet during impact depended on the surface structures with *S* calculated for the respective substrate topography.

As noted above, line friction, fluid inertia or bulk liquid viscosity can each be the limiting factor for spreading^21,25,26,36,41^, depending on the process and the material properties. In order to determine the relative importance of inertia and line friction^41^, the rate of change of kinetic energy can be estimated as $\rho {U}^{3}{R}^{2}$, the line friction dissipation as ${\mu }_{f}{U}^{2}R$, and the rate of work done by the surface tension as $\gamma UR$ (*ρ* is density, *γ* is liquid-gas surface tension, *R* is length scale taken as droplet radius in droplet spreading, and *U* is the spreading speed). It is found^41^ that for a line friction Ohnesorge number greater than unity, $O{h}_{f}={\mu }_{f}/\sqrt{\rho \gamma R}\gg 1$, line friction will determine the spreading, inertia can be neglected, and the properties of the substrate will enter through *μ*_*f*_. In the opposite case, $O{h}_{f}\ll 1$, line friction can be neglected, the spreading will follow an inertial timescale, and the early spreading will be insensitive to the surface properties. In a similar fashion it can be deduced that bulk viscosity can be neglected in early spreading when ${\mu }_{f} > \mu$.

Even though *S*, the ratio of wet area to projected area, has been successful in quantitatively describing the influence of the substrate roughness^36,40,42^, the mechanism by which the hindering happens has not been made clear. Also, for substrate structures that are highly asymmetric, as for instance a sawtooth shape, we would expect a direction dependence on the spreading, which cannot be captured by the simple area ratio.

In this paper we will describe a series of experiments of droplet spreading on sawtooth-like surface structures with well-defined pitches and angles. A qualitative ‘toy model’ is formulated and applied to the geometries of the substrate structures, and predictions are compared to experiments. This allows us to describe the actual mechanism by which the structures hinder spreading, and how features of the structure will affect the spreading speed.

## Theory

### Spreading model

One way to introduce the line friction into a mathematical description is to formulate a coupled Navier-Stokes Cahn-Hilliard (NSCH) equation and allow for dissipation at the contact line.

We will not repeat the NSCH equations here, but suffice it to say that the crucial boundary condition (Eq. (4)^32^ for example) can be used^43^ to derive a relation between static contact angle *θ*_*e*_, dynamic contact angle *θ*, surface tension *γ*, line friction *μ*_*f*_, and contact line speed *U*, as:

3 $$\begin{array}{c}U=\frac{{\boldsymbol{\gamma }}}{{{\mu }}_{{f}}}\frac{(\cos \,{{\theta }}_{{\boldsymbol{e}}}-\,\cos \,{\theta })}{\sin \,{\theta }}\frac{3}{2\sqrt{2}}\end{array}$$

Equation (3) is similar in form to other equations that introduce a line friction, notably the MKT theory^28,29^. The precise expression here is the one that emerges from the NSCH equations^43^, including the factor 3/(2√2 sin*θ*). This allows us to identify the values of the line friction coefficients *μ*_*f*_ with those used previously in comparison between numerical simulations and experiments^31,32,41^.

The dynamic spreading is influenced not only by line friction but can also be controlled by inertia^21^ or bulk viscosity, or the slow viscously dominated final spreading that follows Cox-Voinov or Tanners laws^25,26^. Equation (3) is not intended to be a comprehensive model of wetting in itself; when used as a boundary condition in a full numerical simulation, the Navier-Stokes equations account for the importance of inertia or bulk viscous dissipation^31,32,41^, giving the correct behavior for inertial or viscous spreading.

Equation (3) can be used to determine the spreading speed for the case when spreading is controlled by line friction, i.e. early spreading with $O{h}_{f}={\mu }_{f}/\sqrt{\rho \gamma R} > 1$ and ${\mu }_{f} > \mu$ and inertia and bulk viscosity can be neglected^25,26,41^. In our experiments the latter condition is always satisfied.

### Derivation of a toy model

In this section, we will derive a qualitative description of the passage of a contact line over a substrate topography, in order both to be guided to a practical tool for prediction, and to elucidate the mechanism for hindered spreading. The strategy is to assume Eq. (3) to hold at the surface, include the microscopic shape variations, and estimate the time for the contact line to pass over a feature of the substrate.

**Figure 1:**
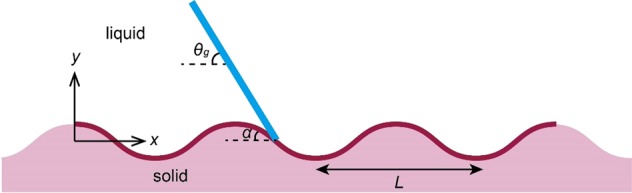
Sketch of a contact line advancing from left to right over a corrugated surface.

The first assumption made in order to allow us to formulate a toy model is that the inclination of the liquid interface is given by the globally observed apparent dynamic contact angle *θ*_*g*_, and that it is unchanged down to the substrate surface as sketched in Fig. 1 considering a sinusoidally corrugated surface. This may seem crude but, as discussed in detail^28^, we notice that when line friction is important, i.e. the line friction Ohnesorge number $O{h}_{f}={\mu }_{f}/\sqrt{\rho \gamma R} > 1$, the dynamic contact angle is allowed to deviate significantly from the static contact angle. For a 1 mm droplet of water on glass or silicon oxide *Oh*_*f*_ is of order unity. However, on a structured surface with micrometer features, an *Oh*_*f*_ based on the structure length scale will be 10 or larger. We interpret this to mean that the liquid interface will not curve significantly when viewed on the microscopic scale as long as this is small enough to make the corresponding *Oh*_*f*_ ≫ 1. In the notation of Fig. 1, the angle *α* between the local substrate surface and the horizontal thus varies as the contact angle passes over the structure. As discussed above, the globally observed apparent contact angle *θ*_*g*_ is, within this qualitative model, assumed to be unchanged as the liquid interface approaches the surface.

Noticing in Eq. (3) that the speed *U* is always along the local solid surface, we rewrite Eq. (3) as

4 $$\begin{array}{c}{\mu }_{f,flat}\frac{ds}{dt}\,\sin ({\theta }_{g}-\alpha )=\gamma (\cos \,{\theta }_{e}-\,\cos ({\theta }_{g}-\alpha ))\end{array}$$

Here we let *s* denote a curvilinear coordinate that follows the surface shape, and *ds*/*dt* is thus the contact line speed, along the surface. The dynamic contact angle *θ* has been replaced by *θ*_*g*_ − *α*, i.e. the local dynamic contact angle assuming that the interface does not curve significantly on the microscopic substrate scale. As expected, the speed is zero when the local dynamic contact angle is equal to the equilibrium angle *θ*_*e*_. *μ*_*f*,*flat*_ denotes the local line friction characteristic of the combination of liquid and solid material, taken to be the same as that which would be obtained on a prefectly flat surface of the same material. Also note that we have replaced the numerical factor $3/2\sqrt{2}=1.061$ by unity, in order to expedite the calculations.

In order to analyze this, we assume the shape to be given by $h(x)={h}_{0}\,\sin \,kx$ with ${h}_{0}k\ll 1$. This allows us to expand all quantities in $\varepsilon ={h}_{0}k\ll 1$, i.e.

$$\frac{ds}{dt}\,\sin ({\theta }_{g}-\alpha )=\frac{dx}{dt}\,\sin \,{\theta }_{g}-\frac{dy}{dt}\,\cos \,{\theta }_{g}=(\sin \,{\theta }_{g}-\varepsilon \,\cos \,{\theta }_{g}\,\cos \,kx)\frac{dx}{dt}$$

$$\cos ({\theta }_{g}-\alpha )=\,\cos \,{\theta }_{g}+\varepsilon \,\sin \,{\theta }_{g}\,\cos \,kx-\frac{1}{2}{\varepsilon }^{2}\,\cos \,{\theta }_{g}{\cos }^{2}kx+O({\varepsilon }^{3})$$

Inserting these expressions into Eq. (4) and expanding for small *ε* yields finally an equation for *dx/d*t, from which the time *T* required for the contact line to advance one wavelength *L* = 2*π*/*k* can be calculated:

$$T=\frac{{\mu }_{f}}{\gamma }L\frac{\sin \,{\theta }_{g}}{\cos \,{\theta }_{e}-\,\cos \,{\theta }_{g}}(1+\frac{{\varepsilon }^{2}}{4}\frac{2+{\cos }^{2}{\theta }_{g}-3\,\cos \,{\theta }_{e}\,\cos \,{\theta }_{g}}{{(\cos {\theta }_{e}-\cos {\theta }_{g})}^{2}})$$

Note that if *ε* = 0, we recover the spreading speed on a flat plate, and that the increase in time required to advance the contact line is proportional to *ε*^2^, and multiplies a function of *θ*_*g*_ and *θ*_*e*_. As expected, the time becomes singular when *θ*_*g*_ and *θ*_*e*_ are equal, i.e. when equilibrium is reached and the motion stops.

We will now put this in the form of an equivalent flat plate, with the substrate structure represented as a correction factor for the line friction. For a flat plate with line friction *μ*_*f*_ = *Sμ*_*f*,*flat*_, where *μ*_*f*,*flat*_ denotes the surface-chemistry line friction obtained on a prefectly flat surface of the same material, the time to travel the distance *L* is

$$T=\frac{{\mu }_{f,flat}}{\gamma }L\frac{\sin \,{\theta }_{g}}{\cos \,{\theta }_{e}-\,\cos \,{\theta }_{g}}$$

Equating this with the time for the structured surface we obtain an expression for *S*:

$$S=1+\frac{{\varepsilon }^{2}}{4}\frac{2+{\cos }^{2}{\theta }_{g}-3\,\cos \,{\theta }_{e}\,\cos \,{\theta }_{g}}{{(\cos {\theta }_{e}-\cos {\theta }_{g})}^{2}}=1+\frac{{\varepsilon }^{2}}{4}{G}_{s}({\theta }_{e},{\theta }_{g})$$

**Figure 2:**
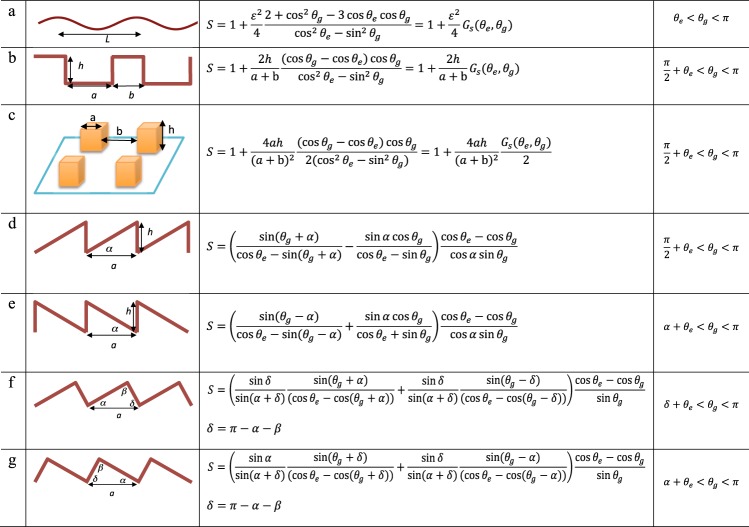
Expressions for *S* and range of *θ*_*g*_ for different structures. The contact line moves from left to right in all cases.

It should be noted that if the function ${G}_{s}({\theta }_{e},{\theta }_{g})$ would be unity, $S=1+{\varepsilon }^{2}/4$, which is the ratio of the total wet length, i.e. the arclength over a period, divided by its projected length, giving a measure of how wrinkled the surface is. And the shape of the function ${G}_{s}({\theta }_{e},{\theta }_{g})$ will be different for different substrate topographies. We find typically that within the framework of a toy model, the function *G*_*s*_ can often be taken as a constant of order unity, and a representative value is obtained for *θ*_*g*_ taken as the midpoint of the intervals in the fourth column of Fig. 2. Here that would be ${G}_{s}({\theta }_{e},({\theta }_{e}+\pi )/2)$ which is equal to 2.00 for a perfectly wetting case.

**Figure 3:**
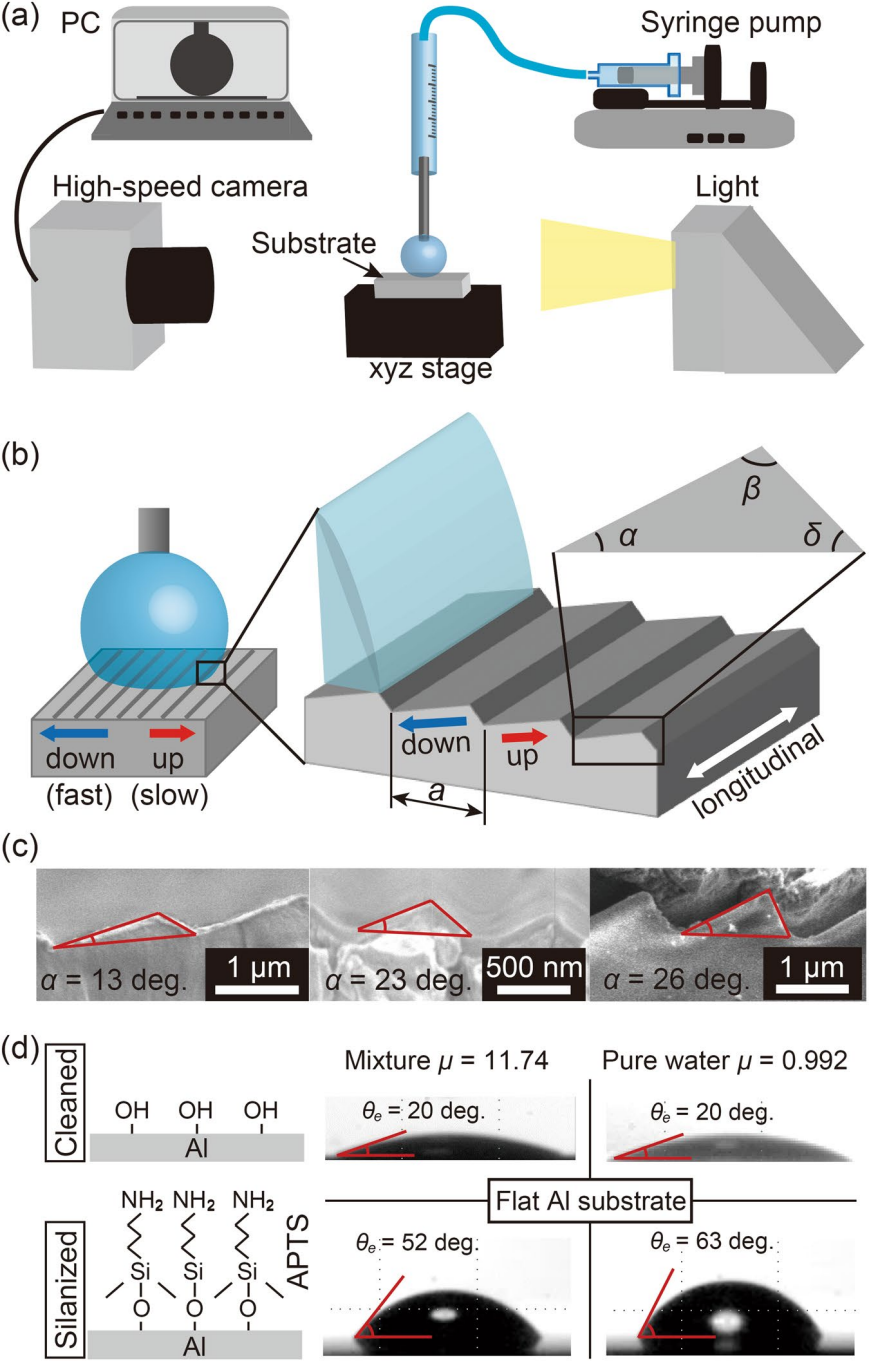
(**a**) The setup of the dynamic wetting experiment. (**b**) A sketch of doplet spreading with definition of spreading directions and geometry. (**c**) Scanning electron micrographs of the sawtooth substates. (**d**) Silanization of the substrate surface for wettablity variation (left) and the resulting static contact angles obtained over the flat Al substrate (right).

The same scheme can now be applied to study other substrate structures. Six additional structures are sketched in Fig. 2, together with their corresponding expressions for *S*, defined as above as a factor multiplying the line friction *μ*_*f*_ = *Sμ*_*f*,*flat*_ on an equivalent smooth substrate (For structures in rows b and c, see Supplementary Theory). Rows d-g in Fig. 2 shows two-dimensional asymmetric sawtooth geometries. Throughout Fig. 2, the contact line is assumed to be traveling from left to right, and we will in the following be referring to cases d, f as ‘up’ and cases e, g as ‘down’. Refer also to the sketch in Fig. 3(b). In case d, we expect the vertical faces to be slowest as the local dynamic contact angle will be lower there and thus closer to *θ*_*e*_ than on the uphill part. We do not expect the contact line to pin as long as *θ*_*g*_ > *θ*_*e*_ + *π/2* but the advancement down the vertical face will be very slow when *θ*_*g*_ is close to *θ*_*e*_ + *π*/2. Looking at the expression for *S*, we see that the first term on the right hand side becomes unity if the inclination angle *α* = 0, and the second term becomes zero, thus recovering the flat plate case. This expression is not readily interpreted as a surface ratio, which was the case in a-c.

As noted above, *S* varies modestly away from the endpoints of the interval in column 4 in Fig. 2. For case d, with a perfectly vertical face, *S* = 2.99 for a perfectly wetting case (*θ*_*e*_ = 0), and an angle *α*  = 23 degrees, taken in the middle of the interval, at *θ*_g_ = 3*π*/4.

Case e is the same geometry as in d, but now mirrored so that the contact line will travel down the sloping part and up on the vertical face. This configuration is less prone to pinning, occurring at *θ*_g_ = *α* + *θ*_e_. The vertical face is now the fastest, and any hindrance will be for the most part due to the slower motion on the inclined face. The value of *S* for a similar perfectly wetting case as above, and the same inclination angle *α* = 23 degrees, is *S* = 1.63 This is less than half the value obtained for the ‘up’ configuration in case d, showing that the effective line friction in this direction is less than half of that for the ‘down’ configuration. The contact line will overall be faster going ‘down’ (e) than going ‘up’ (d).

Cases f and g show a more general saw tooth geometry, with arbitrary inclinations of all faces. To maintain the notions of ‘up’ and ‘down’, we will label the angles in the triangle so that *α* < *δ*, with *β*  = *π* − *α* − *δ*. For the same angle of *α*  = 23 degrees as above, but with a more open angle at the ridge, *β* = 90 degrees, and a perfectly wetting liquid, the values of *S* at the midpoints of the angle ranges, are 1.87 for the ‘up’ (row f) and 1.43 for the ‘down’ (row g) configurations. As might be expected, the ‘up’-case still shows the largest hindrance, but the difference between ‘up’ and ‘down’ is less.

Finally, it should be pointed out that all the expressions for *S* in Fig. 2 are given in terms of angles or nondimensional ratios of lenghts in the microstructure. That is to say that the absolute length scale (for instance *a* in rows d–g) in the microstructures does not influence the results, as long as the surfaces are geometrically similar. This would hold as long as the substrate patterns are small compared to the droplet size and large compared to molecular length scales.

## Results

The spontaneous droplet spreading experiments were carried out using the setup illustrated in Fig. 3(a). Asymmetric sawtooth surface structures with different angles and wettabilities were realized by surface functionalizing grated substrate coated with aluminum. Three different structures were used with *α* of 13, 23, and 26 degrees as shown in Fig. 3(c), and the values of *α*, *β* and *δ* of the substrates were summarized in Table 1. Hereafter we denote each substrate with the values of *α*, as in “13-degrees substrate”. As illustrated in Fig. 3(d), we treated the surfaces in two ways: one is for hydrophilic surface by ultrasonic and UV ozone treatments, and the other is APTS((3-aminopropyl)triethoxysilane) functionalization for partial wetting surface. The measurements were mainly done with purified water and a mixture of purified water, glycerol, and ethanol (1:2:1 in weight ratio) with lower surface tension and higher viscosity compared to that of purified water. Detailed properties of the liquids are given in Table 2. While it is possible in general that a mixture could segregate during the process^44^, we have not encountered anything indicating that this would occur here. The obtained average static contact angles *θ*_*e*_ on the flat aluminum substrate is shown in the right hand of Fig. 3(d). The static contact angles on the different types of satwooth substrates are given in Supplementary Table S1.

**Table 1:** Angles and length of the sawtooth surface.

*α*	*β*	*δ*	*a*
13°	115°	52°	1.67 μm
23°	112°	45°	0.82 μm
26°	87°	67°	1.67 μm

**Table 2:** Fluid properties.

Property	Water	Mixture
Density *ρ* (kg/m^3^)	997	1075
Viscosity *μ* (mPa s)	0.992	11.74
Droplet radius *R* (mm)	0.39	0.40
Surface tension *γ* (N/m)	0.072	0.0365
*Oh*	0.00593	0.0893
*μ*_*f*_ on Al -OH (mPa s)	25	200
*Oh*_*f*_ on Al -OH	0.149	1.596
*μ*_*f*_ on Al -APTS (mPa s)	—	200
*Oh*_*f*_ on Al -APTS	—	1.596

A crucial property will be the line friction coefficient *μ*_*f*,*flat*_, which is obtained by performing a spreading experiment with each of the two liquids on a flat surface with each of the two surface treatments. The value of *μ*_*f*,*flat*_, for each case is then obtained by performing matching numerical simulations and identifying the *μ*_*f*,*flat*_ value that gives the best fit to the experiments. The results are summarized in Table 2.

Using these values of *μ*_*f*,*flat*_ the line friction Ohnesorge number $O{h}_{f}={\mu }_{f,flat}/\sqrt{{\varrho }\gamma R}$ can be calculated for our experiments. As noted above, we expect the spreading to be dominated by the inertia when *Oh*_*f*_ < 1, and dominated by the line friction when *Oh*_*f*_ > 1. As seen in Table 2, *Oh*_*f*_ here ranges from 0.149 for water on ozone-cleaned surface, to 1.596 for viscous mixture on APTS functionalized surface. The values of *μ*_*f*,*flat*_ are quite different for the two liquids, and on the two surface treatments, so that the range of *Oh*_*f*_ both below and above unity is covered. It should be noted that the ordinary viscous Ohnesorge number $Oh=\mu /\sqrt{{\varrho }\gamma R}$ given in Table 2 is significantly less than unity for both liquids. This implies that a capillary driven flow such as droplet oscillations is primarily inertial for both liquids; even if the mixture has approximately ten times larger viscosity than that of water, which is not enough to make the capillary bulk flow viscous.

**Figure 4:**
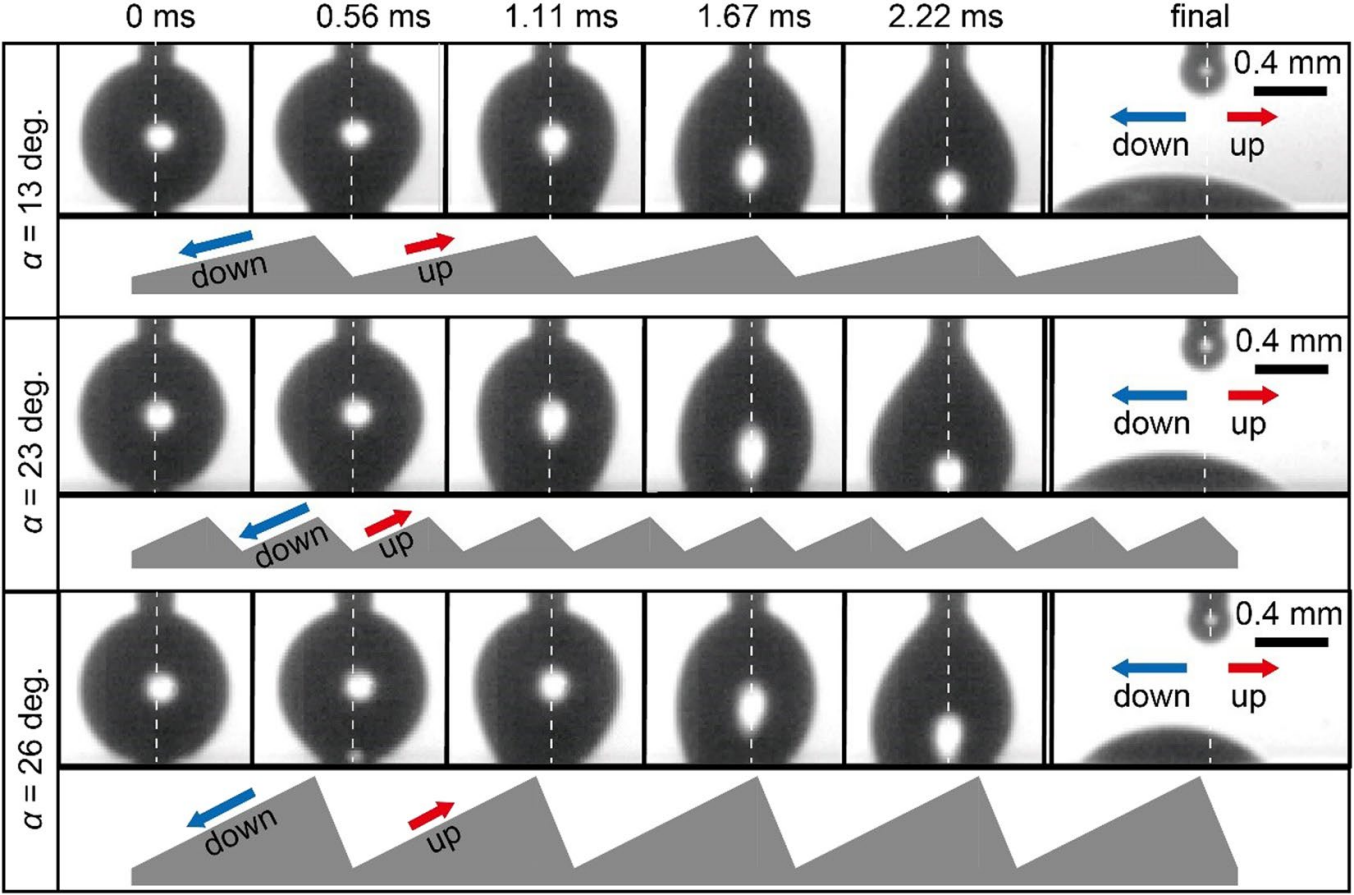
Experimental observation of spreading of the mixture on the three different surfaces. Ridges extend into the plane of the figure, as indicated in the sketch.

Figure 4 shows snapshots of the droplet evolution for the viscous mixture spreading on the hydrophilic substrates, for all three geometries. The droplets are viewed along the ridges of the sawtooth structures, which are illustrated beneath each sequence. In each of the three cases, the droplet is gently brought into contact with the substrate and starts to spread. The left and right sides of each droplet will, due to the peculiarities of the substrate geometry, spread at different speeds giving rise to the asymmetric shapes shown at the intermediate times. This will also result in a net displacement of the droplet after it has detached from the needle and has come to rest (the final snapshots in Fig. 4). The asymmetry of the spreading is largest for the 26-degrees and smallest for the 13-degrees substrate. The slowest spreading always occurs in the direction labelled ‘up’; this refers to the direction where the contact line goes up the longer face making the smaller angle *α* with the horizontal. The ‘down’ direction is characterized by the contact line going down the same long face. Not shown here is the view perpendicular to the sawtooth pattern, where the contact line spreads longitudinally along the ridges. As will be seen below, this spreading is very similar to that on a flat surface. In all cases the droplet footprint is ellipsoidal with a ratio between the major and minor axes which is at most equal to 2. The contact line thus has a significant curvature in the plane of the substrate everywhere.

**Figure 5:**
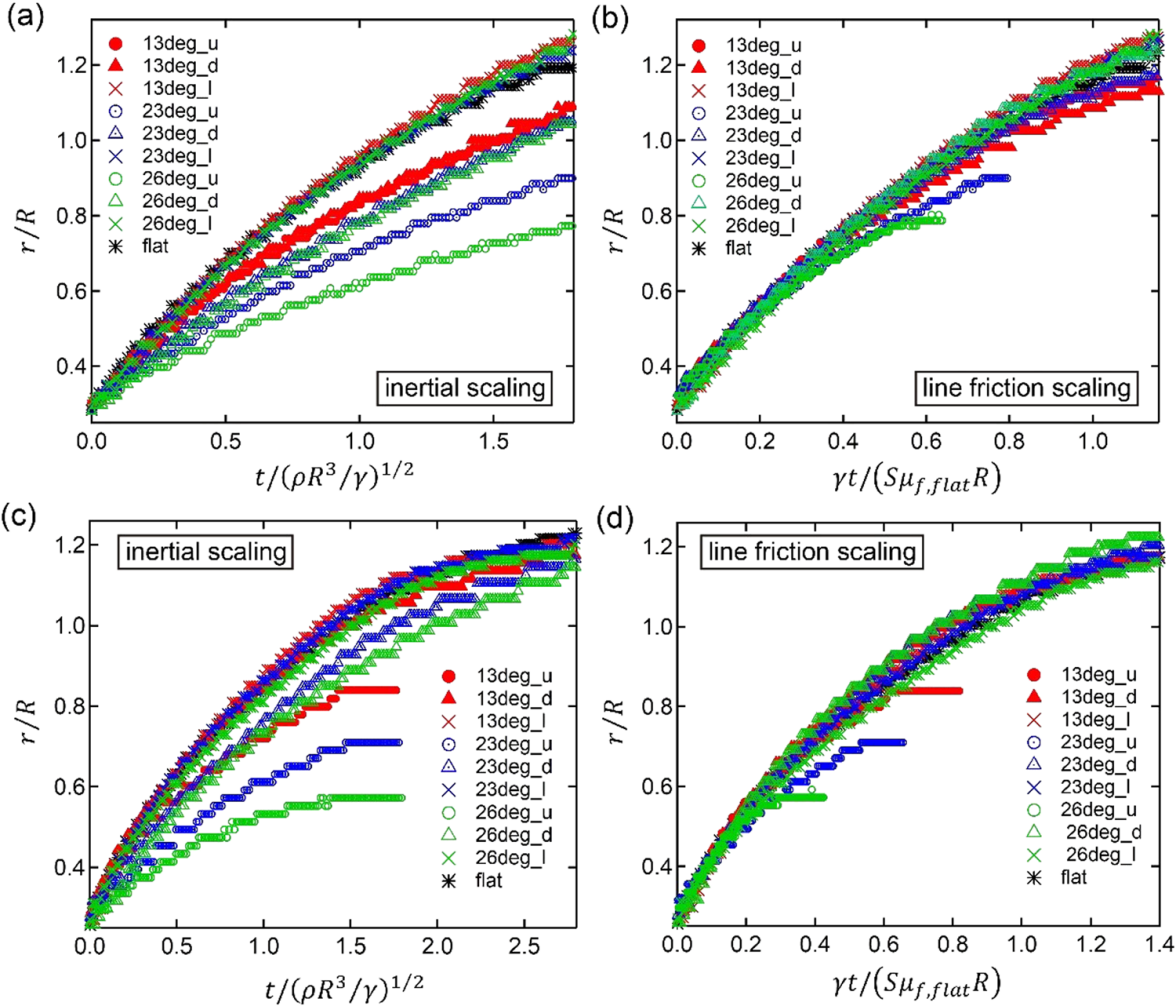
Nondimensional spreading radius of the mixture (*μ* = 11.74 mPa s, *γ* = 36.5 mN/m) on ozone-cleaned surface (*θ*_*e*_ = 20 deg.) for (**a**,**b**) and on APTS functionalized surface (*θ*_*e*_ = 52 deg.) for (**c**,**d**) with line friction *Oh*_*f*_ of 1.596. (**a**,**c**) Inertial timescale, and (**b**,**d**) line friction timescale corrected according to Fig. 1, rows f and g. Symbol shapes of circle, triangle and cross mean the spreading direction to up, down, and logitudinal respectively for 13 deg. (red), 23 deg. (blue), and 26 deg. (green) structures.

Figure 5 shows the positions of the contact lines for the mixture on the three substrates, with the hydrophilic (a),(b) and partial wetting (c),(d) surface treatment. For this case we estimate *Oh*_*f*_ = 1.596 > 1 (see Table 2), and we thus expect the line friction to be limiting the spreading, and the toy model derived above to be applicable. The results are shown as the nondimensional spreading distances *r*/*R* versus time nondimensionalized using an inertial scaling, $\sqrt{\rho {R}^{3}/\gamma }$, where *R* is the initial droplet radius, and *ρ* and *γ* are the density and surface tension of the liquid. As this scaling does not involve any property of the substrates, the timescales are the same for all series in Fig. 5(a), and a plot of the original dimensional data would look the same. It is quite clear that the substrate structures do modify the spreading speeds as there is no collapse of the curves. For each substrate the spreading distance is recorded in the three directions of up (u), down (d), and longitudinal (l). As a reference the spreading on a perfectly flat substrate with the same hydrophilic treatment is also recorded. The first thing to notice is that the longitudinal spreading for all three substrates and the flat spreading are virtually indistinguishable. For the 13-degrees substrate case (red) the ‘down’ and ‘up’ curves are very similar and both about 20% slower than the longitudinal one. For the 23-degrees substrate case (blue), the ‘down’ direction is 20% slower than the flat and the longitudinal, and the ‘up’ direction about 30% slower. The largest asymmetries are visible for the 26-degrees substrate with the ‘down’ direction 20% slower and the ‘up’ 45% slower than the longitudinal spreading.

In order to test the toy models in Fig. 2, the *S* factors listed there have been used to rescale the time according to a line friction time scale $S{\mu }_{f,flat}R/\gamma$. Figure 5(b) shows the spreading data using the expressions for *S* in rows f and g of Fig. 2 for the ‘up’ and ‘down’ spreading, calculated for their respective angles *α*, *β*, *δ*, and *θ*_*e*_. In order to have a single representative number that can be used in the scaling of time, *S* is evaluated at the midpoint of the corresponding interval in column four of Fig. 2. As an example, for the 26-degrees case the *S* values obtained this way for ‘up’ and ‘down’ are 2.06 and 1.50 respectively. The ‘longitudinal’ spreading is assumed to be very similar to that of spreading on a flat surface of the same material, and a value of *S* = 1 is thus used there. In rescaling Fig. 5(b), all time values in the 26-degrees ‘up’ case will thus be divided by *S* = 2.06, and all times in the 26-degrees ‘down’ case by 1.50, and both curves will move to the left. A collapse of all curves, ‘up’, ‘down’, and ‘longitudinal, onto a master curve, would signify that the toy model can perfectly describe how the slowing down of the spreading depends on the substrate geometry. Figure 5(b), does indeed show a very good collapse of all the data onto a single curve. The deviation for the later stages of spreading in the ‘up’ direction on the 23-degrees and 26-degrees substrates (blue and green circles respectively) is due to the pinning of the contact line when it reaches a position of *r*/*R* approximately equal to 0.9 and 0.8 respectively.

The fact that the line friction time scale in Fig. 5(b) does collapse the data, as opposed to the inertial timescale in Fig. 5(a) shows that inertia is of lesser importance, and that line friction determines the spreading, as expected from the value of *Oh*_*f*_ = 1.596 > 1.

Figure 5(c,d) shows the results for the three substrate geometries when they have been made partially wetting, with a *θ*_*e*_ = 52 degrees. With the inertial scaling in Fig. 5(c), the picture is rather similar to that in Fig. 5(a), with the ‘longitudinal’ spreading on all substrates coinciding with the spreading on a flat substrate. This is slightly slower for the partially wetting case compared to the hydrophilic one in Fig. 5(a), but the difference is not great initially. The ‘up’ spreading is slow for all cases, with the 26-degrees geometry the slowest. Figure 5(d) shows the results of rescaling the time in a similar way as in Fig. 5(b), but now using *θ*_*e*_ = 52° in the calculation of the *S*-factors. Again the collapse onto a master curve is quite satisfactory. The deviations for the ‘up’ cases in the later stages are again due to the pinning of the contact lines. On this more hydrophobic surface this pinning happens somewhat earlier, but the overall collapse of the data up to that point is good.

**Figure 6:**
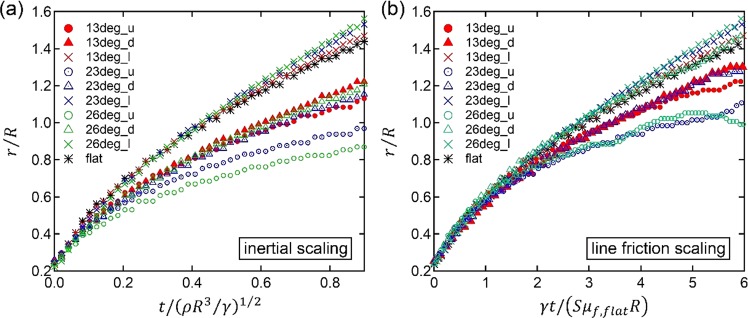
Nondimensional spreading radius of the water (*μ* = 0.992 mPa s, *γ* = 72 mN/m) on ozone-cleaned surface (*θ*_*e*_ = 20 deg.) with line friction *Oh*_*f*_ of 0.149. (**a**) Inertial timescale, and (**b**) line friction timescale corrected according to Fig. 1, rows f and g. The meaning of each symbol shape and color is identical to that of Fig. 4.

Figure 6 shows the results of using water on the hydrophilic surfaces. For water we estimate that *Oh*_*f*_ = 0.149 < 1, and here we should expect that inertia comes in as a factor in hindering the spreading, and consequently that the details of the substrate become less important. Figure 6(a) shows the spreading using the inertial scaling of time, as above. Despite the low value of *Oh*_*f*_, the spreading is still different in the different directions, with all the longitudinal spreading aligning with spreading on a flat substrate, and the ‘up’ spreading the slowest. However, in the early spreading for r/R < 0.4, we see a collapse in the curves in Fig. 6(a) which is not present in Fig. 5(a,c).

Rescaling time using the line friction time scale for each substrate produces Fig. 6(b) which still shows a better collapse onto the curve for spreading on the flat surface than the inertial scaling, but with a larger spread than in Fig. 5(b,d). This however is to be expected as the toy model does not account for inertia. Despite the comparatively low value of *Oh*_*f*_ = 0.149, the line friction scaling still collapses the data better than the inertial scaling. In the limit of decreasing *Oh*_*f*_ we expect the initial spreading to become independent of the substrate geometry, i.e. all curves for up, down and longitudinal to collapse using the inertial scaling, until the static advancing angle is approached for each case. We conclude that the value of *Oh*_*f*_ = 0.149 for the case in Fig. 6 signifies that this is an intermediate parameter range where both line friction and inertia hinder the spreading. For an even lower value of *Oh*_*f*_ (i.e. larger drop, lower surface tension, lower contact line friction) we expect that the dependence on the substrate structure would be lower and that the inertial time scale would collapse the curves better for early spreading^41^, in keeping with experiments showing inertial spreading^21^.

We tried spreading experiments using pure water on the partially wetting APTS modified surfaces. However, the spreading in this case is not in the Wenzel state, but that air is trapped in the grooves of the pattern, creating an entirely different spreading scenario, outside the scope of the present model (See Supplementary Results).

## Discussion

Throughout the results, we see that rescaling time according to the toy model consistently reduces the spread of the data, and we conclude that the geometric expressions for *S* in Fig. 2 do capture the essential influence of the geometry on the spreading speed for cases where *Oh*_*f*_ > 1, and still partially for *Oh*_*f *_$\lesssim$ 1.

The reason for the slowing down is the long time that the contact line has to spend on those faces where the local dynamic contact angle becomes closest to the static contact angle *θ*_*e*_, thus reducing the driving force. It should be noted that the slowing down that we see in our experiments is not due to momentary pinning on edges and ridges. All through the advancement that local angle is greater than *θ*_*e*_, and the speed is determined by the local *μ*_*f*,*flat*_ via Eq. (4).

This is thus primarily a question of kinematics: the contact line will spend more of its time on the slow parts of the structure, and even on a symmetric structure where the contact line passes equal distances over fast and slow faces, the quick passage over the fast parts will not make up for the slow parts.

The observation that the factor *S* could be interpreted as the ratio of wet area to projected footprint area for symmetric substrate topographies, we at this point regard as fortuitous. It is the case also for symmetric structures that not all faces are equally important; the most adverse faces will be responsible for the slowing down. Still, the wet to footprint area ratio seems to capture this well for symmetric shapes.

It should also be noted that in cases where the total line friction, including the geometric factors described here, is not large enough to dominate the spreading, i.e., when *Oh*_*f*_  ≪ 1, the spreading will be determined by the total inertia of the drop or the viscous dissipation in the bulk, and the substrate roughness will not influence the spreading.

In conclusion, it is shown that the speed is significantly reduced for spreading in the directions across the ridges of the asymmetrically sawtooth shaped microstructured surfaces, with the greatest reduction when the contact line traverses the more gently sloping face of the ridge going up, and the steepest going down. The toy model that was constructed estimates the local speed of the contact line from the time required for the contact line to travel across the different parts of the structure, limited by a local flat surface line friction and the local departure of the contact angle from its static value. The reasonable agreement between the predictions from the toy model and the experimental results suggests that this is valid as a qualitative description.

## Methods

### Preparation of sample

The surfaces were cleaned by ultrasonic in acetone for 15 minutes and treated by UV ozone for 10 minutes prior to spreading experiments for hydrophilic cases. Also the designated substrates are functionalized following a standard silanization procedure with (3-aminopropyl)triethoxysilane (APTS) in vapor phase for partial wetting cases. The silanization procedure forms a uniform monolayer and thus does not alter surface structures at micro- nor nano-scales.

### Set up of spreading experiment

The desired substrate was set under the thin needle (needle gauge: 33 G, 90°) of which the outer diameter is 0.18 mm. The distance between needle tip and the substrate was fixed to be 0.8 mm. The liquid was slowly delivered to the needle by the syringe pump (KDS 120) at the rate of 0.04 ml per hour, and the quasi-static sphere droplet grew larger. As soon as the droplet touched the substrate with a small enough speed, the spreading started and was observed from the horizontal direction along to the ridges with a high speed camera (Phantom VEO710L, Vision Research Inc., 54000 fps). A lamp (HVC-SL, Photoron) was used to provide lighting with sufficient intensity. After the measurements, the images were digital-processed to extract the time histories of spreading radius. For one substrate, the measurements were repeated three times and spreading radius histories were ensemble-averaged.

## Supplementary information

Supplementary Information
