# Supplementary material for: Revealing How Topography of Surface Microstructures Alters Capillary Spreading
Source: Sci Rep. 2019 May 24;9:7787. doi: 10.1038/s41598-019-44243-x (PMC6534613; doi:10.1038/s41598-019-44243-x)
Supplement: Supplementary file 1 — Supplementary Information [file 41598_2019_44243_MOESM1_ESM.pdf]

Supplementary Information

Revealing How Topography of Surface Microstructures Alters Capillary Spreading

Yaerim Lee[1], Naoto Matsushima[1], Susumu Yada[2], Satoshi Nita[1], Takashi Kodama[1], Gustav Amberg[2,3], and Junichiro Shiomi[1,*]

[1]Department of Mechanical Engineering, The University of Tokyo, Bunkyo-ku, Tokyo, Japan
[2]Department of Mechanics, Linné Flow Centre, The Royal Institute of Technology, Stockholm, Sweden
[3]Södertörn University, Stockholm, Sweden
(*e-mail: shiomi@photon.t.u-tokyo.ac.jp)

Supplementary Theory

Row b in Table 1 (in the manuscript) shows the result for square two-dimensional ridges. Note that when a droplet is placed on the surface, θ_g will initially be 180 degrees, but will decrease towards the value of the advancing static contact angle, where it is expected to be pinned to the corner of the ridge. In the notation here, that will occur at $\theta_g = \pi/2 + \theta_e$, accounting for the singularity in $G_s(\theta_e, \theta_g)$. The fourth column in Table 1 lists the range in θ_g where the expression S is regular, with the lower limit interpreted as the advancing static contact angle. Away from this singularity, the variation of $G_s(\theta_e, \theta_g)$ with θ_g is modest. For a perfectly wetting fluid ($\theta_e = 0$), $G_s = 2.00$ at $\theta_g = \pi$, and $G_s = 2.41$ at $\theta_g = 3\pi/4$, representative of the initial phase of the spreading. For a partially wetting liquid with $\theta_e = 40$ degrees, $G_s = 3.00$ at $\theta_g = \pi$, and $G_s = 3.71$ at $\theta_g = (\pi/2 + \theta_e + \pi)/2$ (the average of the initial and the pinning angle).

Row c in Table 1 (in the manuccript) shows the results for evenly distributed square posts. This expression for S was obtained as an average of the spreading times over square ridges and a flat plate, weighted with the width of the posts a and their spacing b, respectively. We notice that the dependencies on θ_g and θ_e are given by the same function G_s as for the two-dimensional ridges. In this three-dimensional case we expect the contact line to pin at a lower angle than for the two-dimensional ridges, since it may be temporarily halted at the edge of the top of the post, but as the contact line is pulled along past the post on the bottom surface, the post sides will be wetted from the bottom instead.

For the initial spreading ($\theta_g = \pi$) of a perfectly wetting fluid over posts, $G_s = 2.00$ as noted above, which gives the expression $S = 1 + 4ah / (a + b)^2$. This can be interpreted

as the total wet surface area $(a + b)^2 + 4ah$ divided by the projected footprint area $(a + b)^2$. This was noted in our experiments[1,2] on posts of this kind.

In both the square ridges and the posts (rows b and c of Table 1 in the manuscript), it would be expected that the liquid interface could touch a corner of a post or ridge ahead of the contact line, whenever $\theta_g > \pi/2$. Within the context of this toy model we have not taken all such possibilities into account.

Supplementary Results

The static contact angles on the different types of satwooth substrates with different wettabilities and liquids are given in Table S1. Note that when the water droplet placed on the APTS functionalized sawtooth structures, it makes partial Cassie state. In this more hydrophobic case it becomes clear when looking at the evolution of the contact angles, that the final static value becomes 88 degrees in the longitudinal direction, even though it is found to be 63 degrees on the flat substrate. From this we conclude that the spreading in this case is not in the Wenzel state, but that air is trapped in the grooves of the pattern, creating an entirely different spreading scenario, outside the scope of the present model.

Table S1: Measured static contact angles of substrates with different geometry and wettability.

	Static contact angle [°]							
	Hydrophilic (-OH)				Partial wetting (-O-$(CH_2)_3$-NH_3)			
	Normal		Longitudinal		Normal		Longitudinal	
	Water	Mix.	Water	Mix.	Water	Mix.	Water	Mix.
α = 13°	31	29	13	14	94	69	87	52
α = 23°	37	34	13	13	107	66	88	51
α = 26°	36	34	13	13	103	65	89	51

References

1. Wang, J. Y. *et al*. Surface structure determines dynamic wetting. *Scientific Reports* **5**, 8474 (2015).
2. Nita, S. *et al*. Electrostatic cloaking of surface structure for dynamic wetting. *Science Advances* **3**, e1602202 (2017).
